# Supplementary material for: Direct access to poly(glycidyl azide) and its copolymers through anionic (co-)polymerization of glycidyl azide
Source: Nat Commun. 2019 Jan 17;10:293. doi: 10.1038/s41467-018-08251-1 (PMC6336848; doi:10.1038/s41467-018-08251-1)
Supplement: Supplementary file 1 — Supplementary Information [file 41467_2018_8251_MOESM1_ESM.pdf]

**Supplementary Information**

# Direct Access to Poly(glycidyl azide) and its Copolymers through Anionic (Co-)Polymerization of Glycidyl Azide

Senthil et al.

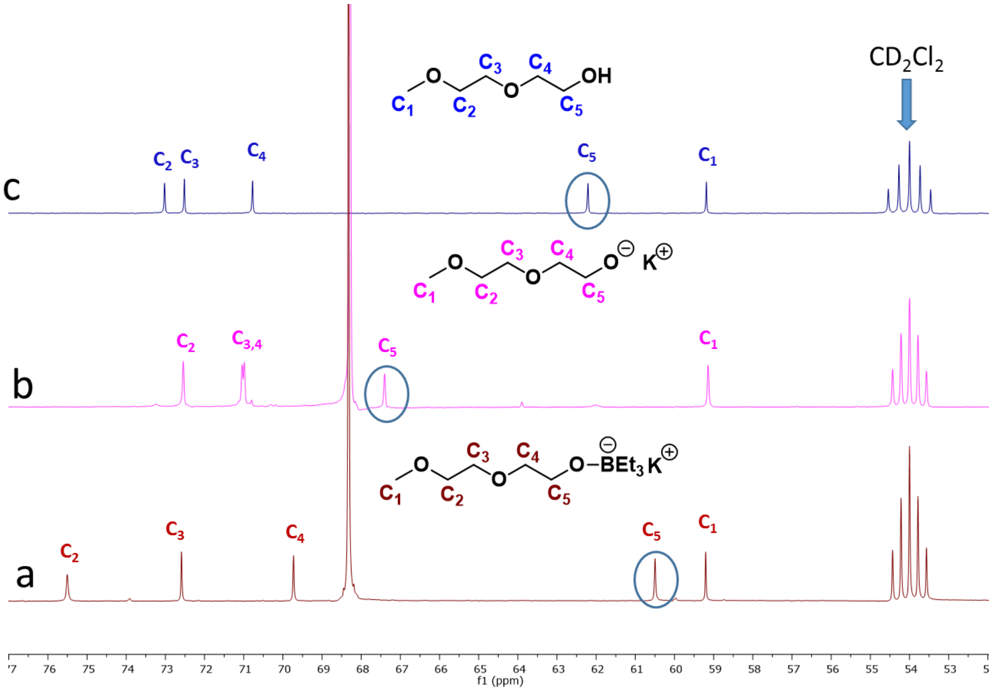


**Supplementary Figure 1.** Model study using $^{13}C$ NMR displaying the formation of TEB-ate-complex at room temperature: **a** corresponds to the ate-complex formed by reaction of $RO^-K^+$ with TEB (ratio = 1:1). **b** and **c** correspond to $RO^-$ $K^+$ and to ROH respectively, where ROH = diethyleneglycol monomethyl ether. The methylene carbon $C_5$ displays a significant upfield chemical shift as compared to the corresponding alkoxide which indicates the formation of an ate-complex.

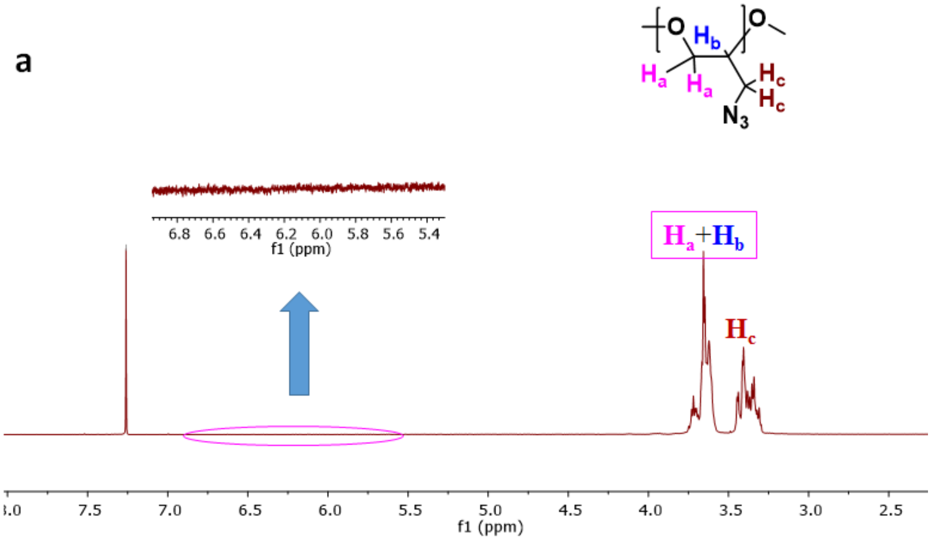


**Supplementary Figure 2a.** $^{1}$H NMR spectrum of the purified PGA sample of entry 1, Table 1. The proton signals between 3.29 and 3.47 ppm correspond to the methylene protons (-$CH_2$-$N_3$) attached to the azido group and the peaks corresponding to methylene (-$CH_2$-O-) and methine (-CH-O-) protons appear at 3.56 -3.78 ppm. The absence of any proton signal between 5.5 and 7.0 ppm indicates that no elimination occurred during the anionic polymerization.

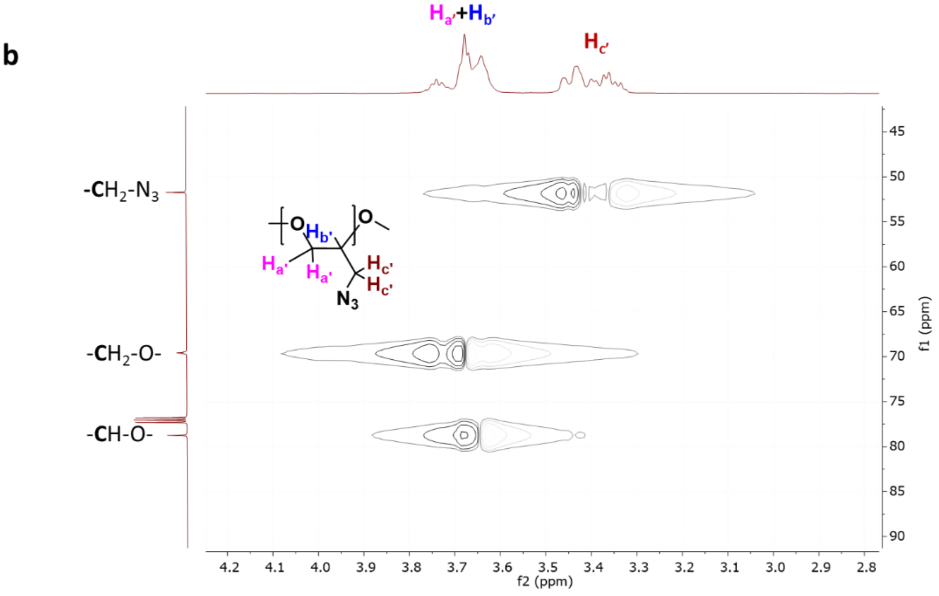


**Supplementary Figure 2b.** HSQC data of the purified PGA sample of entry 1, Table 1. C-H correlations obtained from the 2D NMR characterization unambiguously distinguishes the methylene protons attached to oxygen and to azido functions.

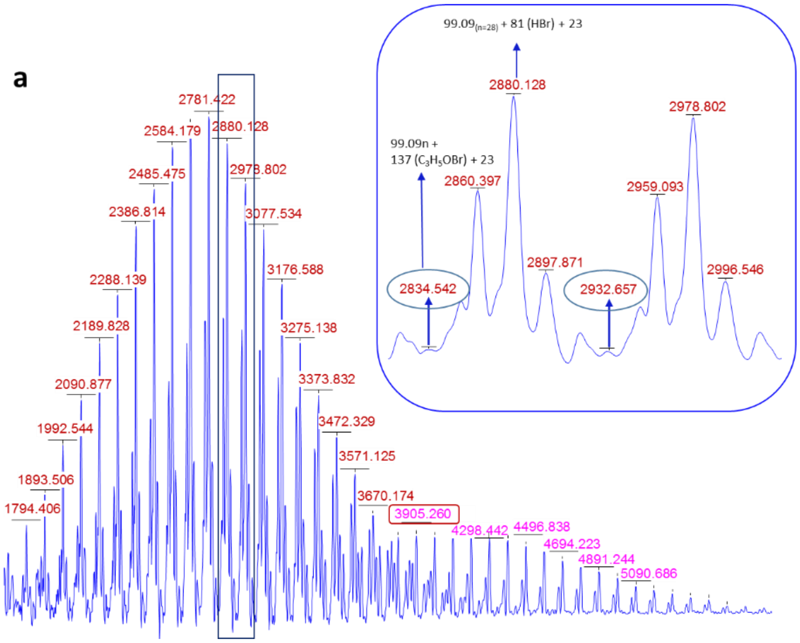


**Supplementary Figure 3a.** MALDI-TOF characterization of the PGA sample corresponding to entry 1,Table 1. Transfer reactions during PGA synthesis occurred via two pathways (Path a: transfer to monomer and Path b: transfer to polymer) both of which result in the formation of branched polymers.

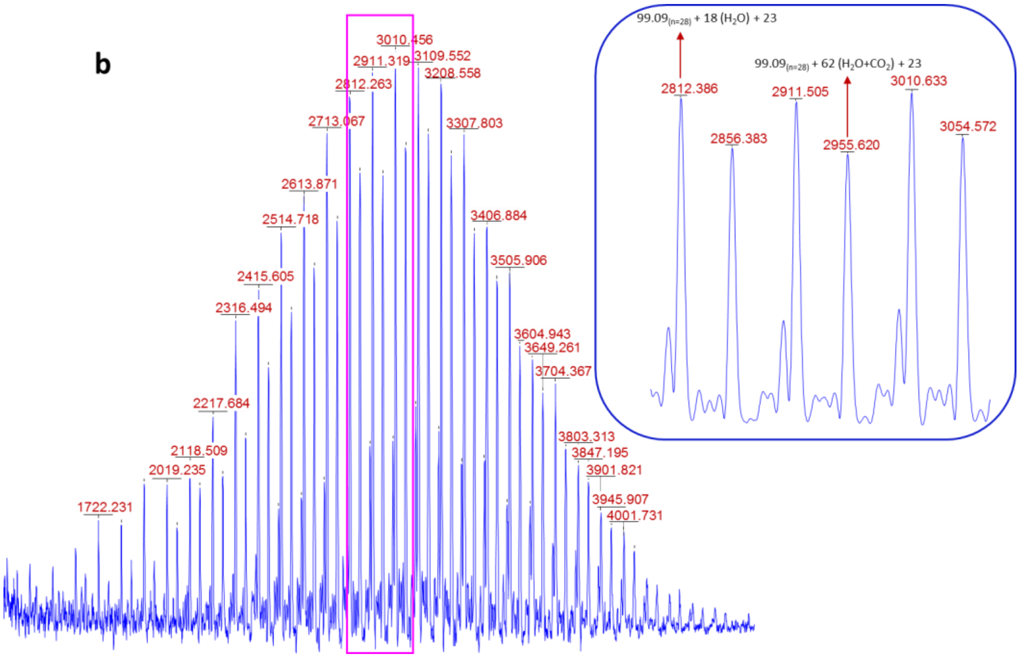


**Supplementary Figure 3b.** MALDI-TOF characterization of the purified PGA-diol sample corresponding to entry 16, Table 1. There are two populations: the signal centered at m/z 2856 [99.09 (28) + 62 ($H_2O$+$CO_2$) +23] corresponds to PGA-diol chains which have a carbonate unit embedded in the polyether chain arising from the carbonate initiator. The signal at 2812 correspond to the PGA-diol chains in which the above mentioned carbonate linkage is missing due to the loss of $CO_2$ during the initiating step.

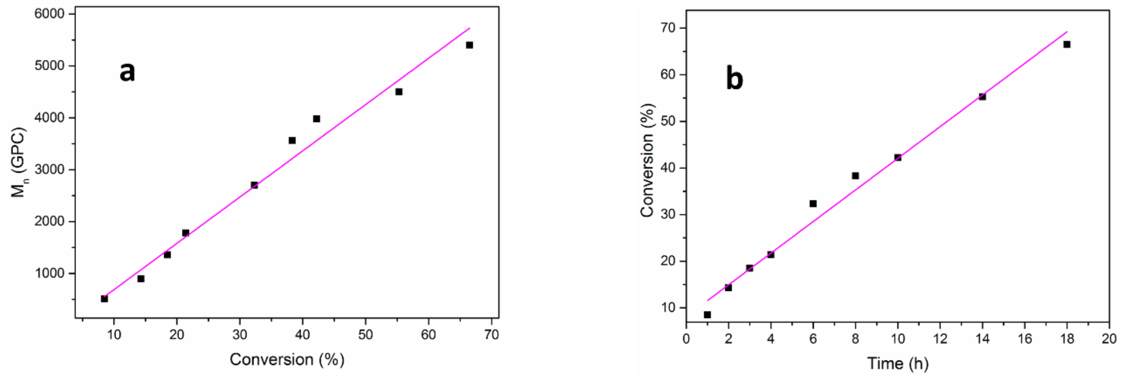


**Supplementary Figure 4.** Kinetic experiments showing the livingness of the GA polymerization using TEB as activator. **a:** displays the linear increase of $M_n$ of PGA vs. GA conversion; **b:** displays the linear increase of conversion vs. time (h).

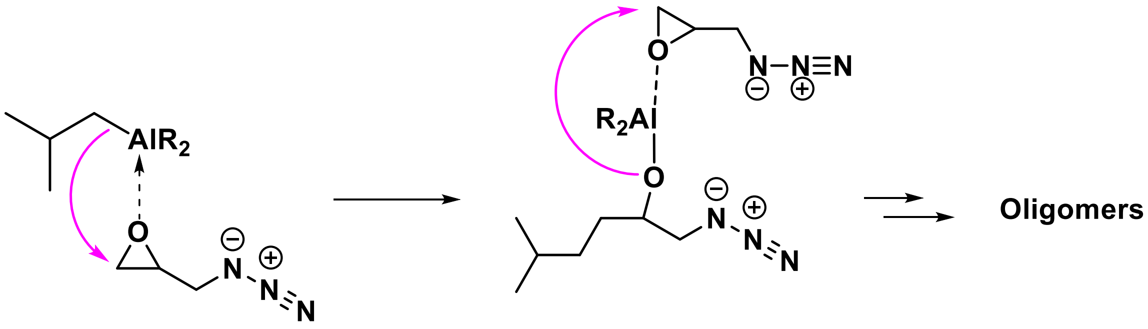


**Supplementary Figure 5.** Mechanism of ring-opening of GA by TiBA. The addition of TiBA (1M in toluene) dropwise at room temperature to GA (ratio = 1:1) leads to its spontaneous reaction with GA, triggering the ring-opening of the latter in an uncontrolled manner.

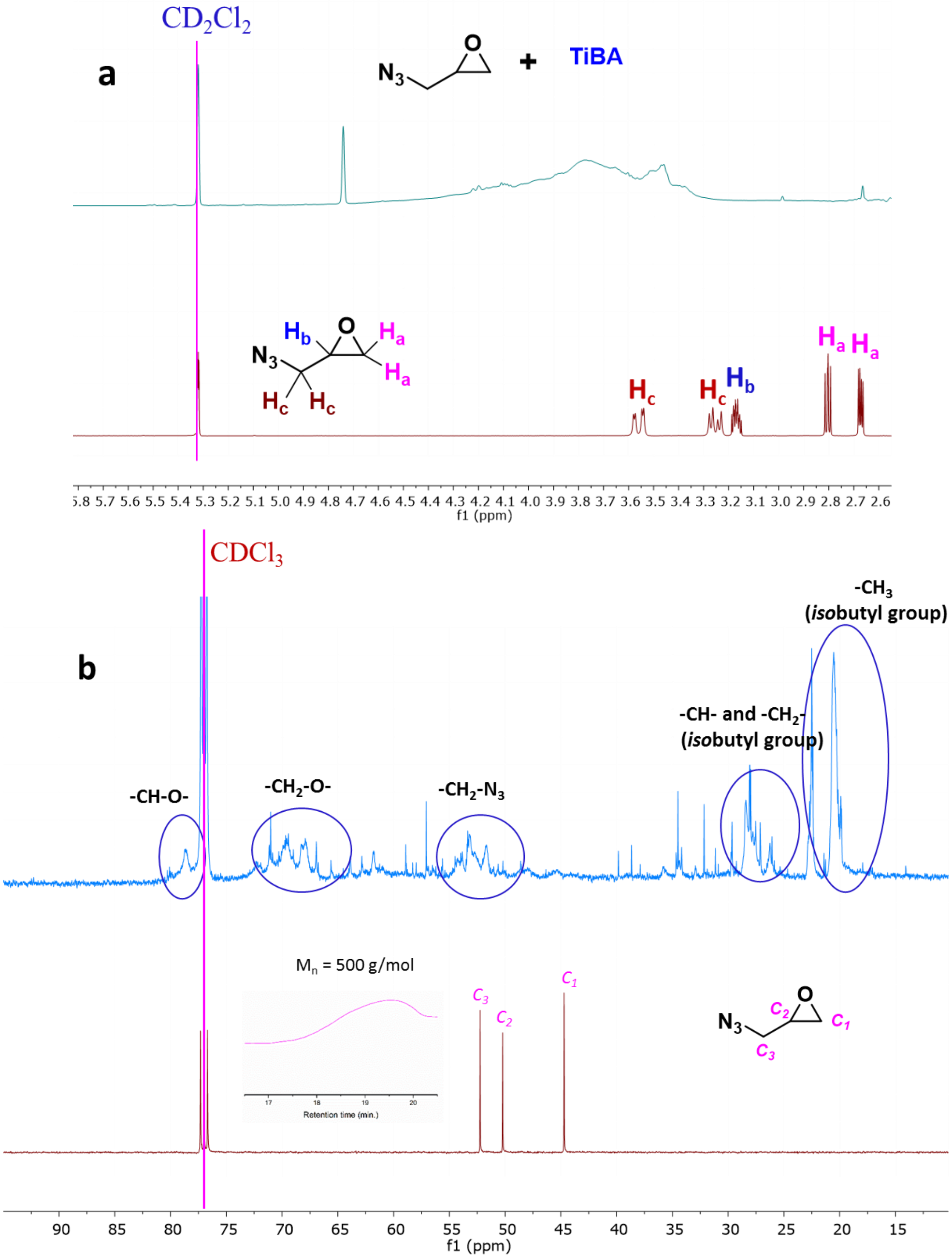


**Supplementary Figure 6. a:** $^{1}$H NMR, **b**: $^{13}$C NMR spectrum and **b**-inset GPC traces of oligomers from upon dropwise addition of TiBA on GA as depicted in Supplementary Figure 5.

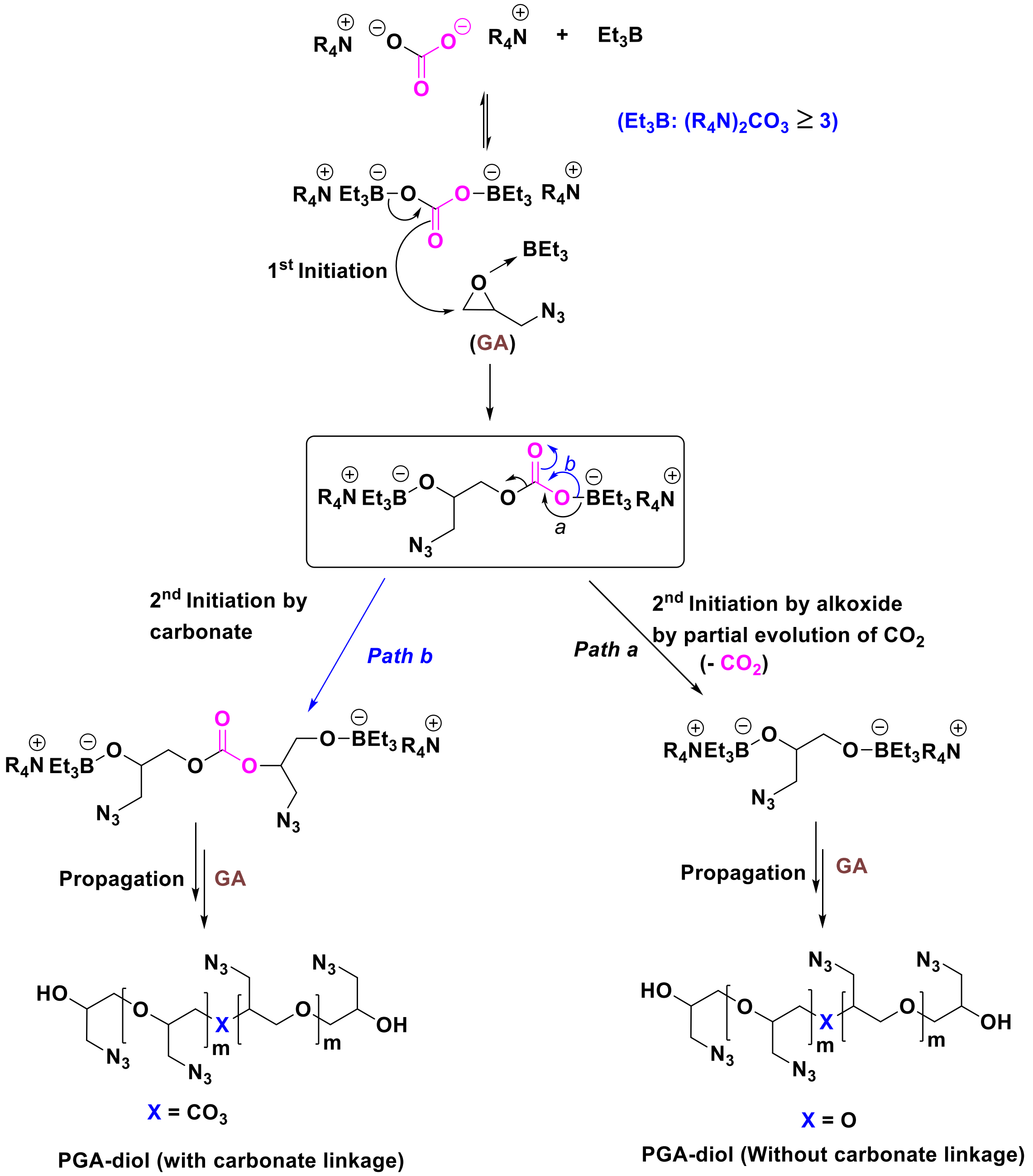


**Supplementary Figure 7.** Scheme detailing the reactions occurring the synthesis of PGA-diol using $(R_4N)_2CO_3$ as bifunctional initiator:

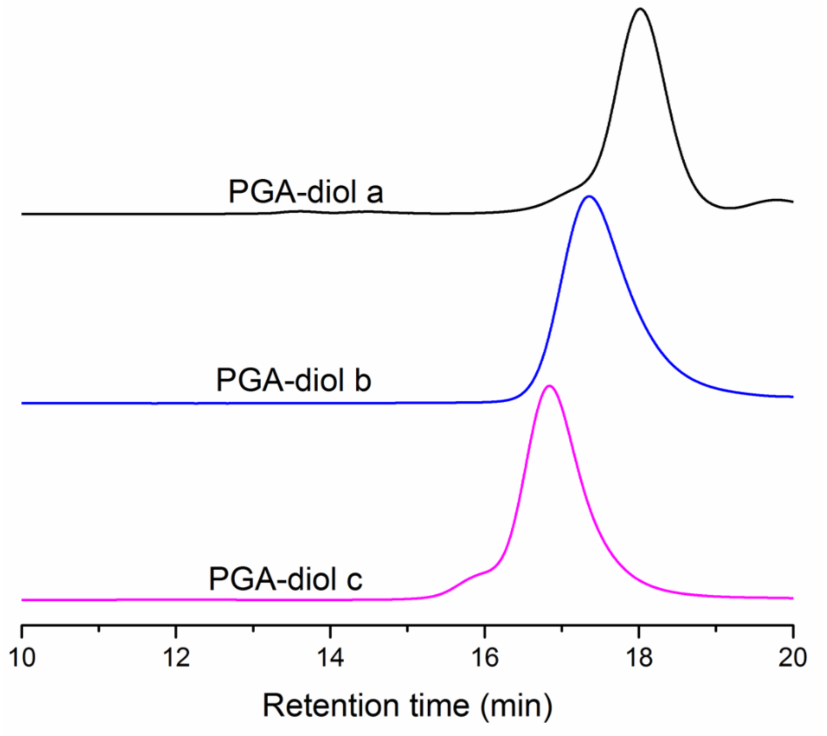


**Supplementary Figure 8.** GPC traces of PGA-diol samples of entries 15-17, Table 1. PGA-diol **a**, **b** and **c** corresponds to entries 15, 16 and 17, respectively. Reaction conditions: PGA-diol **a**: GA/$(Bu_4N)CO_3$/TEB = 20:1:3, **b**: GA/$(Bu_4N)CO_3$/TEB = 25:1:3 and **c**: GA/$(Bu_4N)CO_3$ /TEB = 100:1:5 at 0°C.

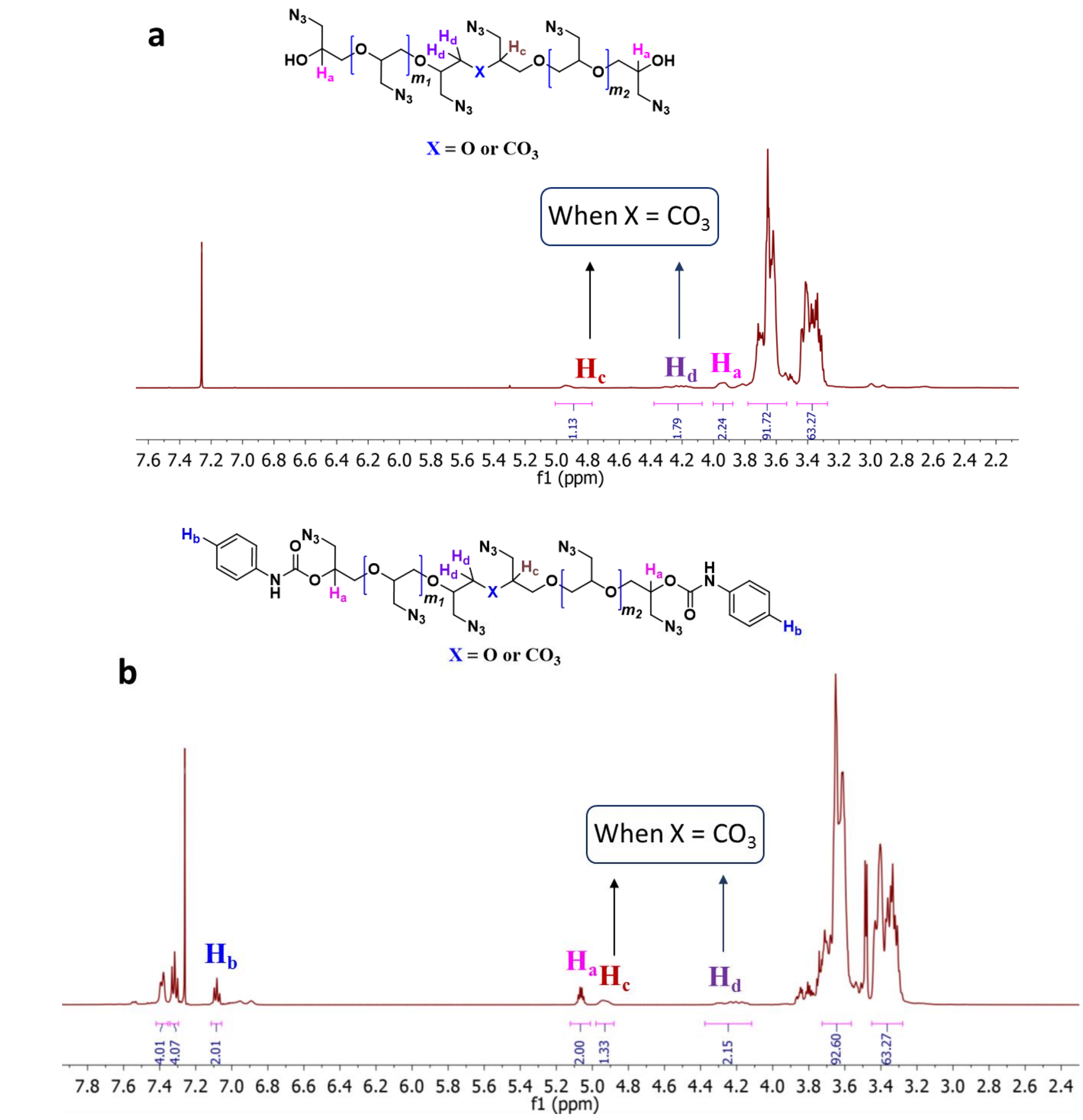


**Supplementary Figure 9. a:** $^{1}$H NMR spectrum of the the PGA-diol sample corresponding to entry 16, Table 1; **b**: urethane adduct formed upon reacting PGA-diol with phenylisocyanate. The $^{1}$H NMR characterization of the diurethane-modified PGA displayed an integral ratio of 2:2 for $H_a$ and $H_b$ which clearly supports the presence of two hydroxyl groups at PGA chain ends.

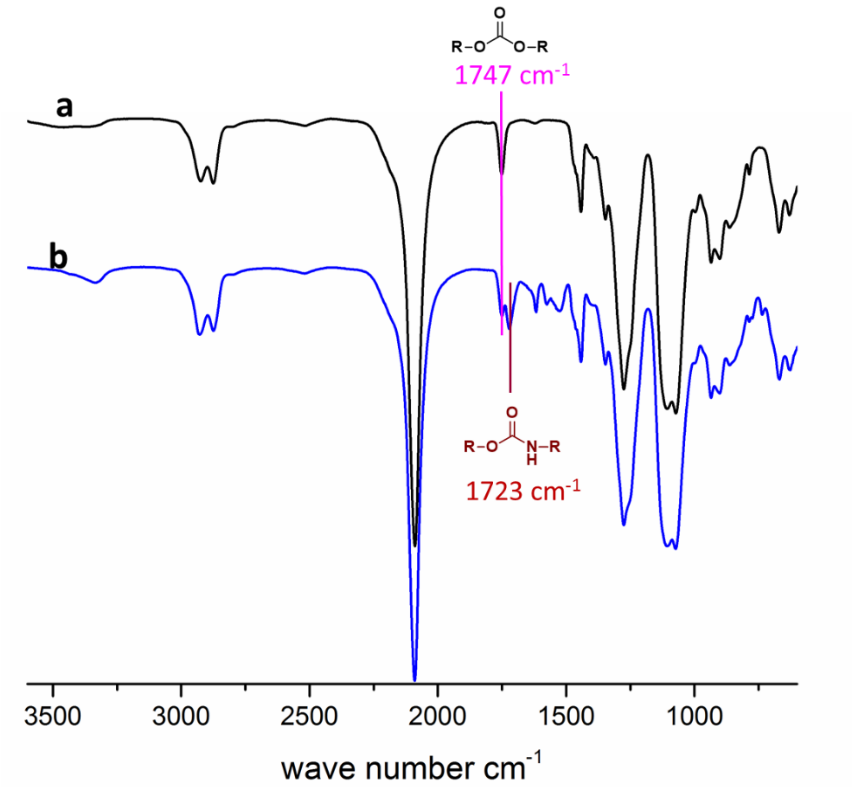


**Supplementary Figure 10. a:** FTIR of the PGA-diol sample corresponding to entry 16, Table 1; **b:** polyurethane sample formed by reaction of PGA-diol with hexamethylene diisocyanate. The appearance of a new FTIR signal at 1723 $cm^{-1}$ in **Figure b** strongly indicates the formation of polyurethane obtained by polycondensation reaction of PGA-diol and hexamethylene diisocyanate.

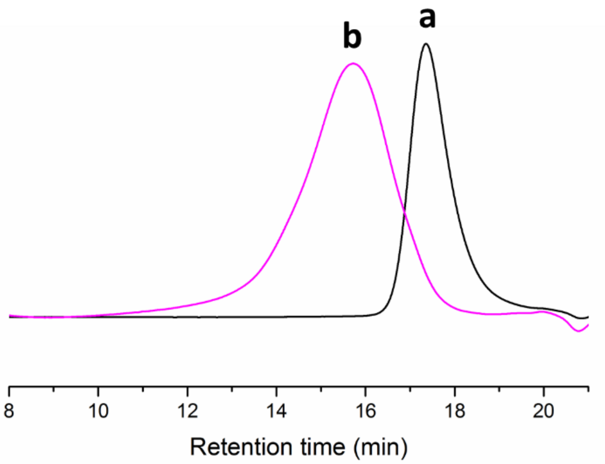


**Supplementary Figure 11. a**: GPC traces of the PGA-diol sample corresponding to entry 16, Table 1; **b:** polyurethane obtained by reaction of PGA-diol (a) with hexamethylene diisocyanate. The polycondensation of PGA-diol with hexamethylene diisocyanate (1.1 equiv) results in the formation of a polyurethane sample which exhibits a number average molar mass of 12.5 Kg/mol, confirming the telechelic nature of the PGA-diol.

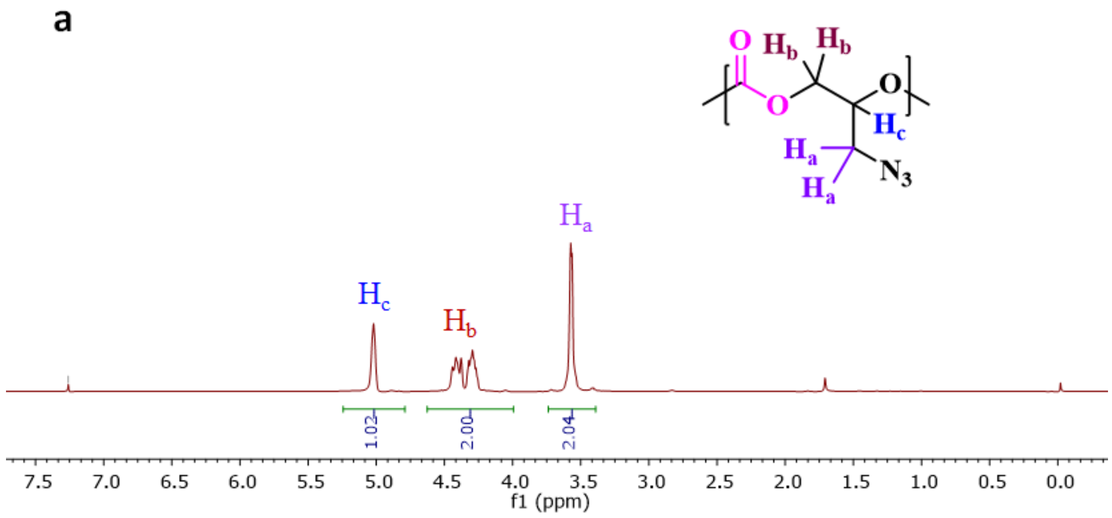


**Supplementary Figure 12a.** $^{1}$H NMR spectrum of the PGAC sample of entry 7, Table 2. The integral ratio of peaks of $\mathbf{H_c}$, $\mathbf{H_b}$, and $\mathbf{H_a}$ (1: 2: 2) indicates an alternating carbonate structure without formation of ether linkages.

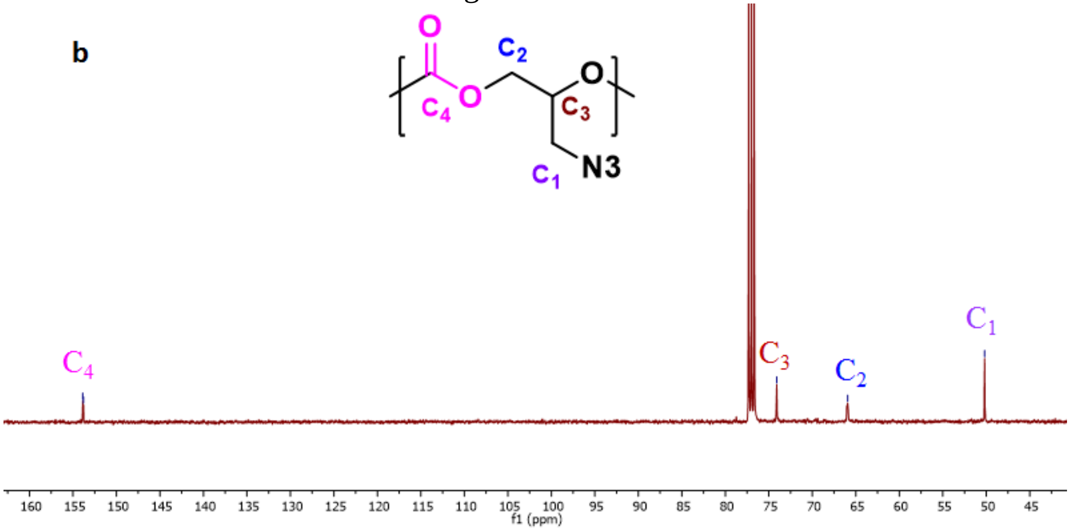


**Supplementary Figure 12b.** $^{13}$C NMR of the PGAC sample of entry 2, Table 2. The absence of ethereal carbons in PGAC supports the alternating nature of the copolymerization and the high carbonate content.

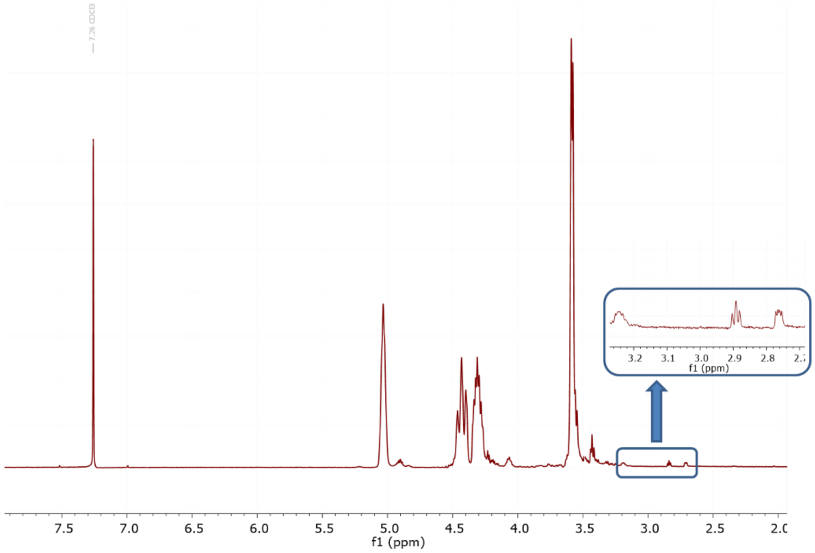


**Supplementary Figure 13.** $^1$H NMR spectrum of the PGAC sample of entry 12, Table 2. The appearance of a residual epoxide signal in this PGAC sample is indicative of transfer to the monomer.

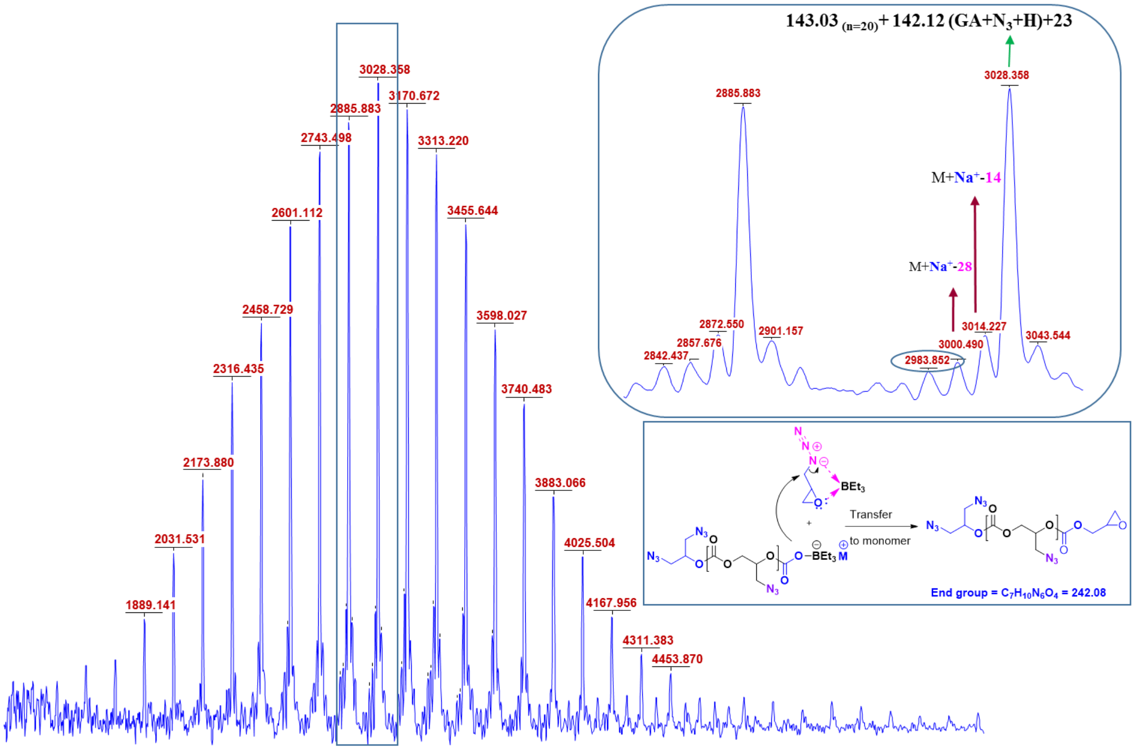


**Supplementary Figure 14.** MALDI-ToF characterization result of the PGAC sample corresponding to entry 12, Table 2. The occurrence of transfer to monomer (see the mechanism in above figure) is evidenced in this MALDI-ToF spectrum which shows the presence of PGAC chains (mass signal encircled at 2983.85 (242.08+ 142.12+ 23) carrying an epoxide end group.

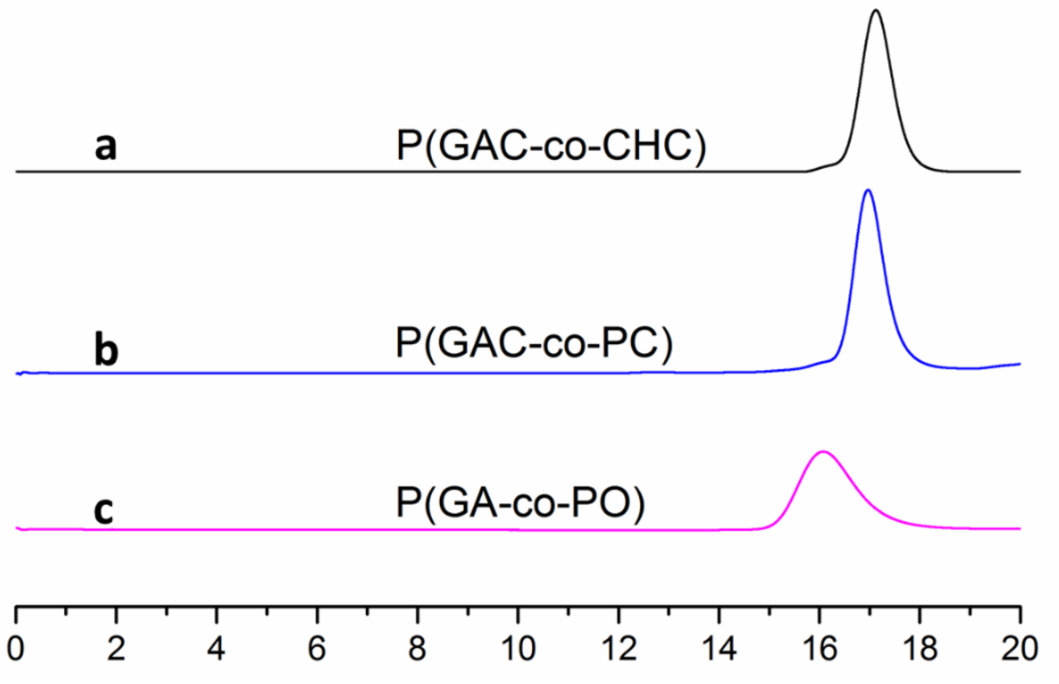


**Supplementary Figure 15.** GPC traces of random terpolymer samples corresponding to entry 1-3, Table 3. The unimodal distribution of the samples **a**: P(GAC-co-CHC), **b**: P(GAC-co-PC) and **c**: copolymer P(GA-co-PO) indicates the random incorporation of GA.

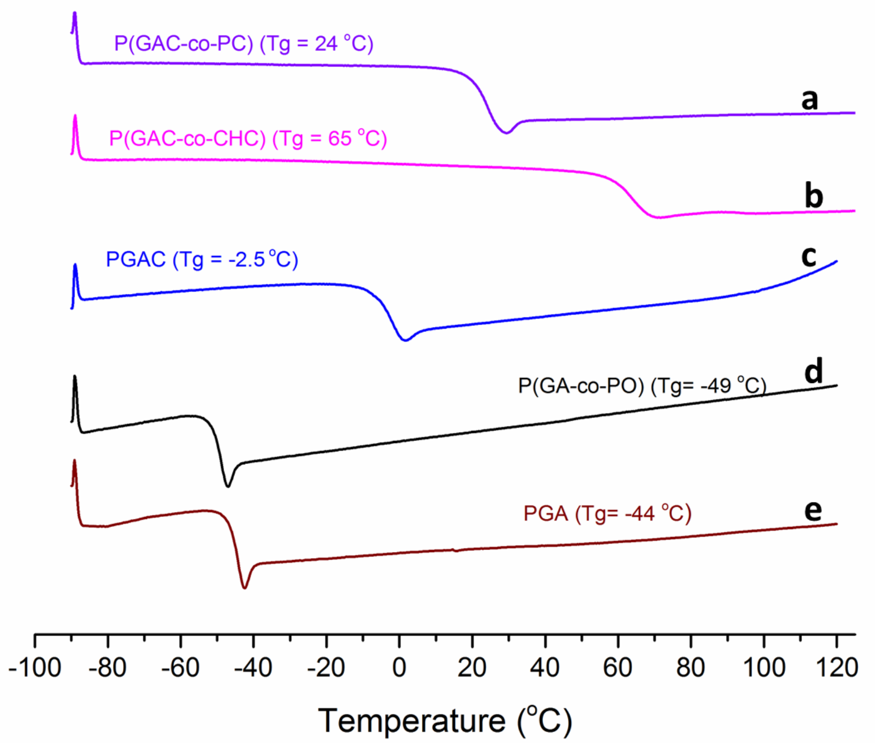


**Supplementary Figure 16.** DSC curves displaying the Tg's of **a**: P(GAC-co-PC); **b**: P(GAC-co-CHC); **c**: PGAC; **d**: P(GA-PO); **e**: PGA. The copolymerization of GA with either PO or CHO is random as evidenced by the DSC data obtained which indicate single Tg for the copolymers obtained. Further, the Tg values obtained are in-between the values of their corresponding homopolymers. For instance, the Tg values of PCHC, PPC and PGAC are ~ 120, ~ 35 and -2.5 $^{o}$C, respectively. The random polycarbonate made of PGAC and PPC exhibits a Tg of 24 $^{o}$C. Similarly, the random polycarbonate made of PGAC and PCHC exhibits a Tg of 65 $^{o}$C. Additionally, PPO and PGA exhibit Tgs of -60 and -44 $^{o}$C and the random copolymer obtained from those polymers shows a Tg of -49 $^{o}$C which clearly demonstrates the random nature of the obtained copolymer.

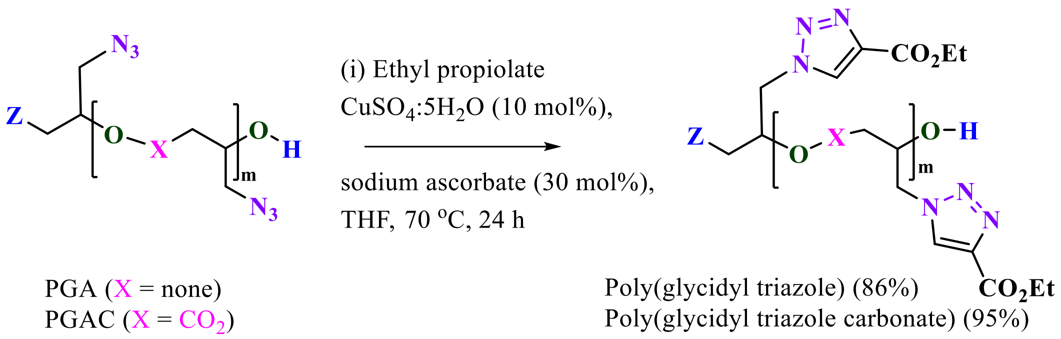


**Supplementary Figure 17.** Click reaction of PGA and PGAC with ethyl propiolate. Reaction conditions: PGA or PGAC (1 equiv.), Ethyl propiolate (2 equiv. with respect to azido function), $CuSO_4.5H_2O$ (10 mol)%, sodium ascorbate (30 mol%), THF, 70 °C, 24 h. Yield: Poly(glycidyl triazole) (86%); Poly(glycidyl triazole carbonate) (95%).

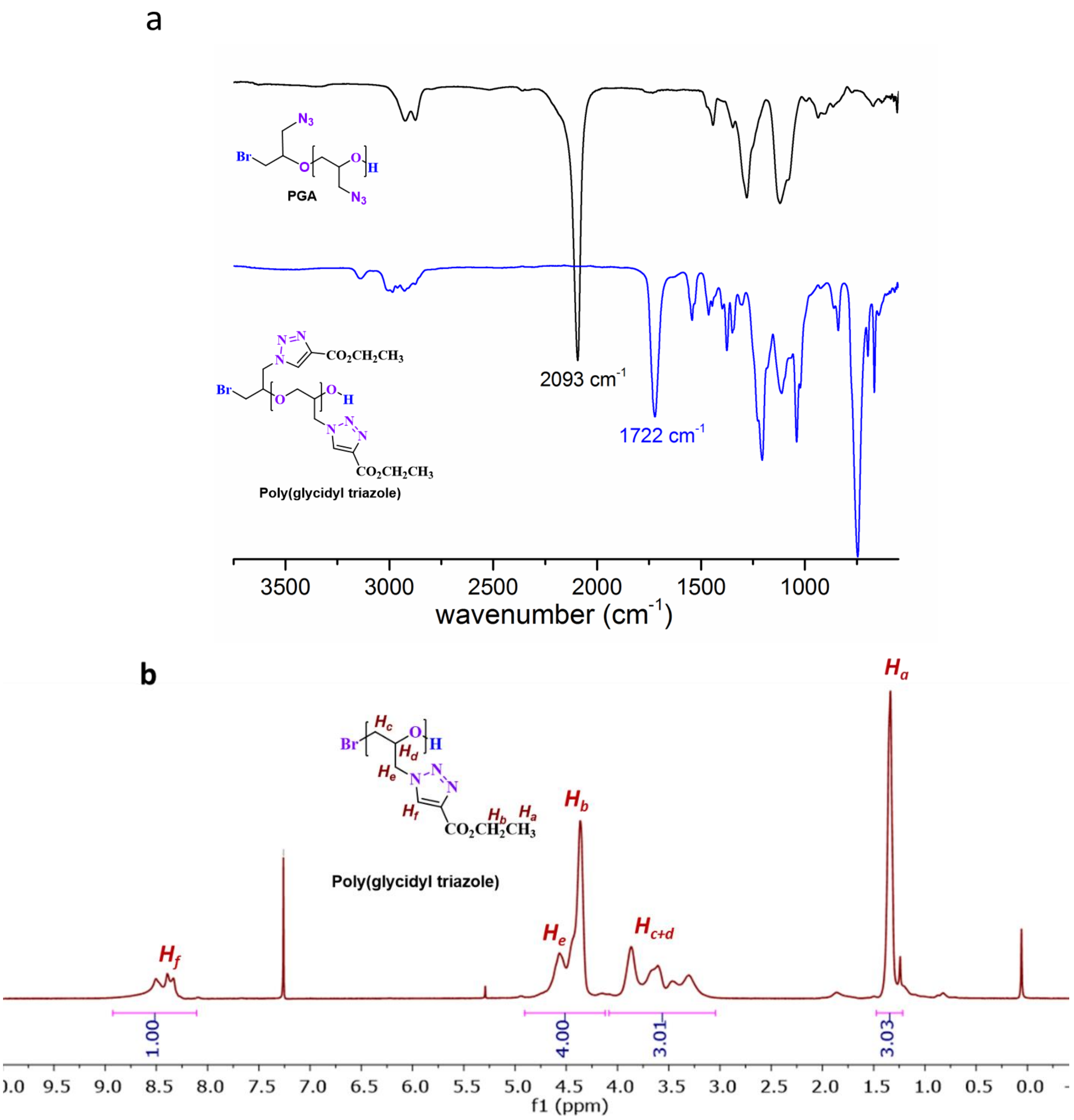


**Supplementary Figure 18.** **a**: FTIR and **b**: $^{1}H$ NMR spectrum of poly(glycidyl triazole). The complete transformation of azido functions in PGA into the corresponding triazole is confirmed by the complete disappearance of the azido signal (2093 $cm^{-1}$) and appearance of a new signal at 1722 $cm^{-1}$ corresponding to the ester groups of ethyl propiolate. It is further supported by $^{1}H$ NMR which displays a typical signal at $\delta$ 8.5 ppm corresponding to the aromatic proton of the triazole moiety.

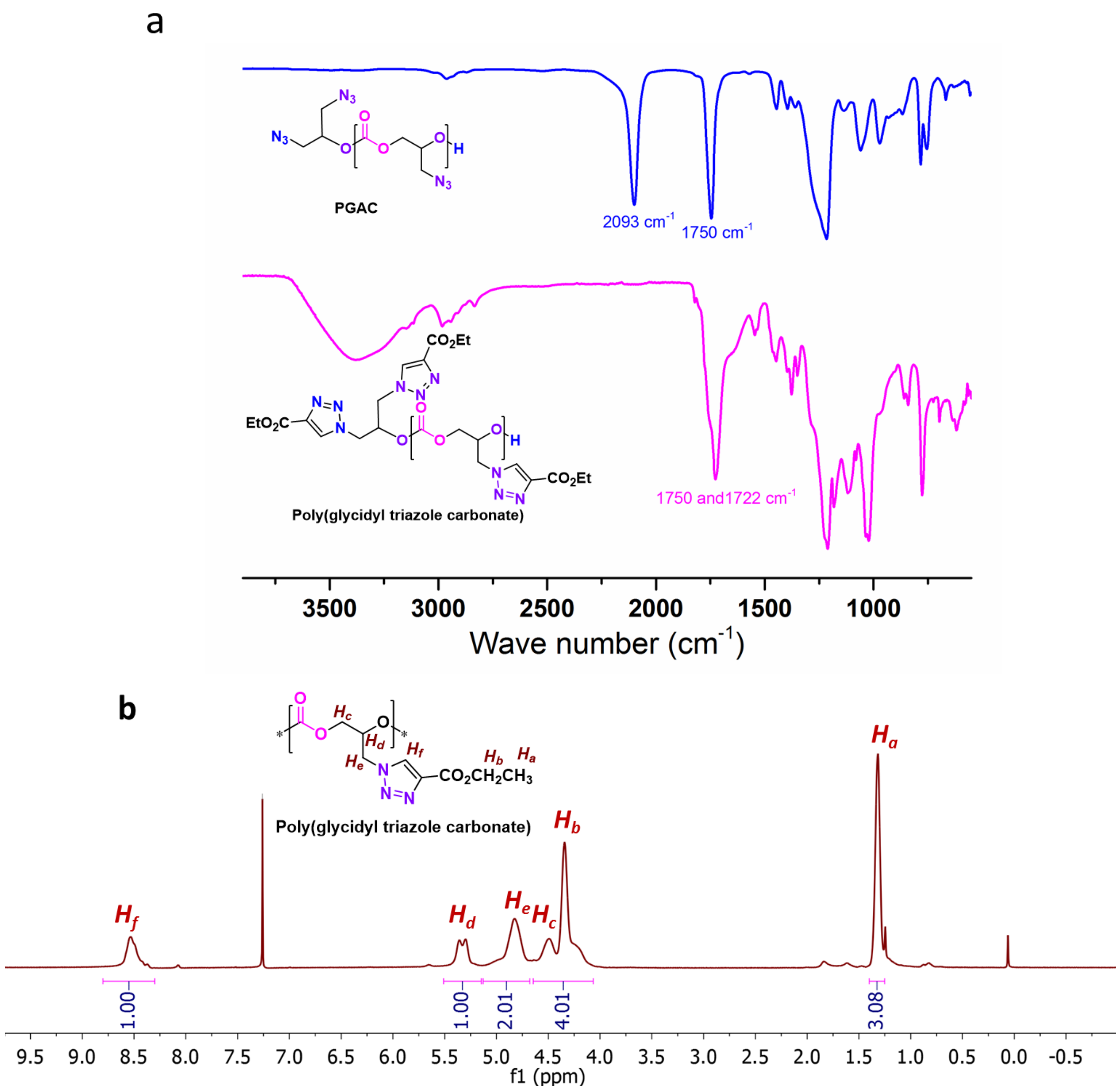


**Supplementary Figure 19. a**: FTIR and **b**: $^{1}H$ NMR spectrum of poly(glycidyl triazole carbonate). The azido function in PGAC is efficiently converted into the corresponding triazole which is confirmed by the complete disappearance of the azido signal (2093 $cm^{-1}$) and appearance of a new signal at 1722 $cm^{-1}$ (merging with carbonate signal) corresponding to the ester groups of ethyl propiolate. This observation is further supported by $^{1}H$ NMR which displays a typical signal at δ 8.5 ppm corresponding to the aromatic proton of the triazole moiety.

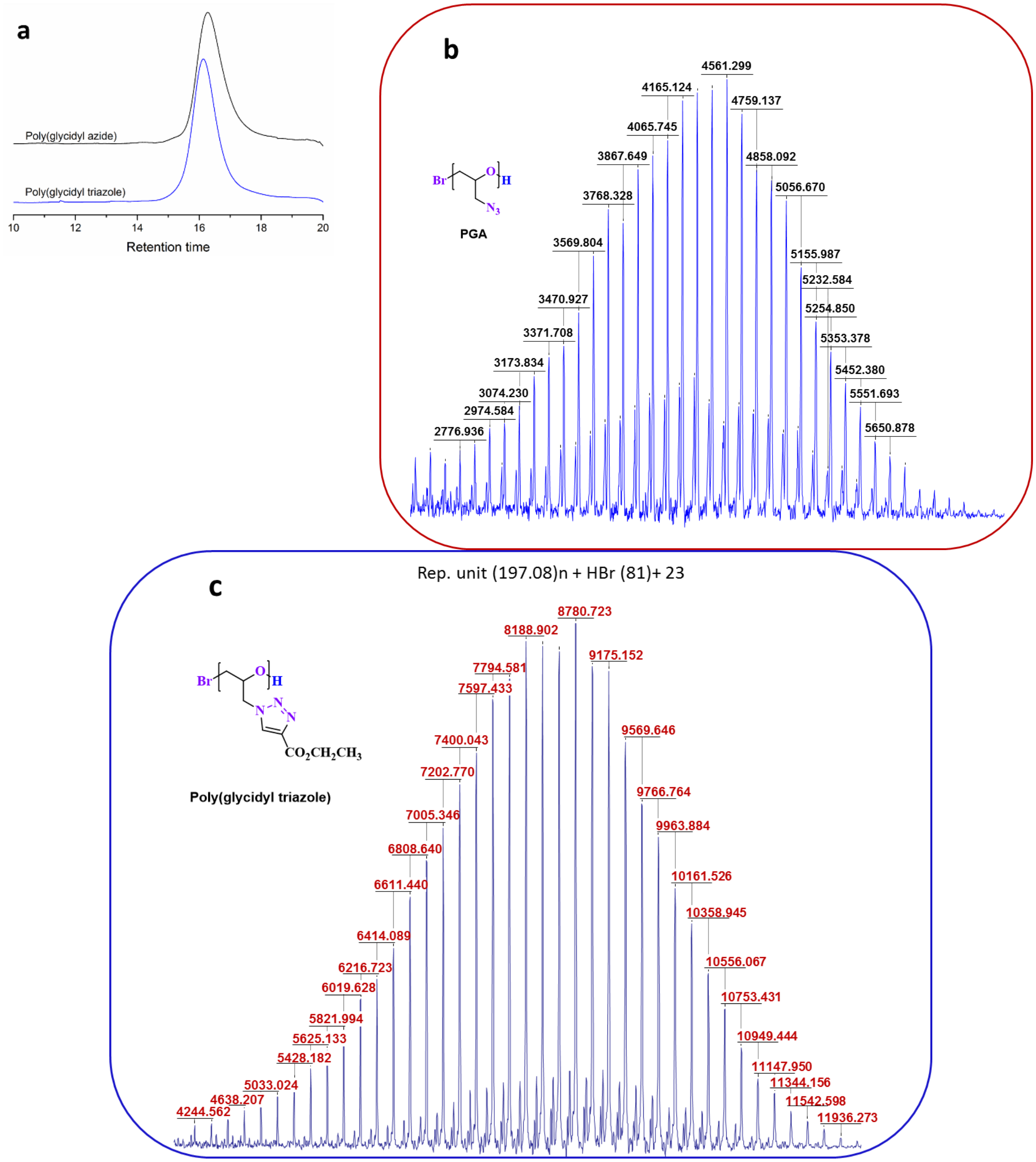


**Supplementary Figure 20. a**: GPC traces of PGA and poly(glycidyl triazole) samples and **b**: MALDI-ToF analysis of a PGA sample and **c**: poly(glycidyl triazole). The main population of **c** corresponds to the poly(glycidyl triazole) carrying bromo (Br) and hydroxyl (OH) end groups [$197.08_{(n)}$ + 81 + 23] with a peak to peak mass difference of 197.08 for one repeating unit, where 81 and 23 are the molar mass of HBr and $Na^+$.

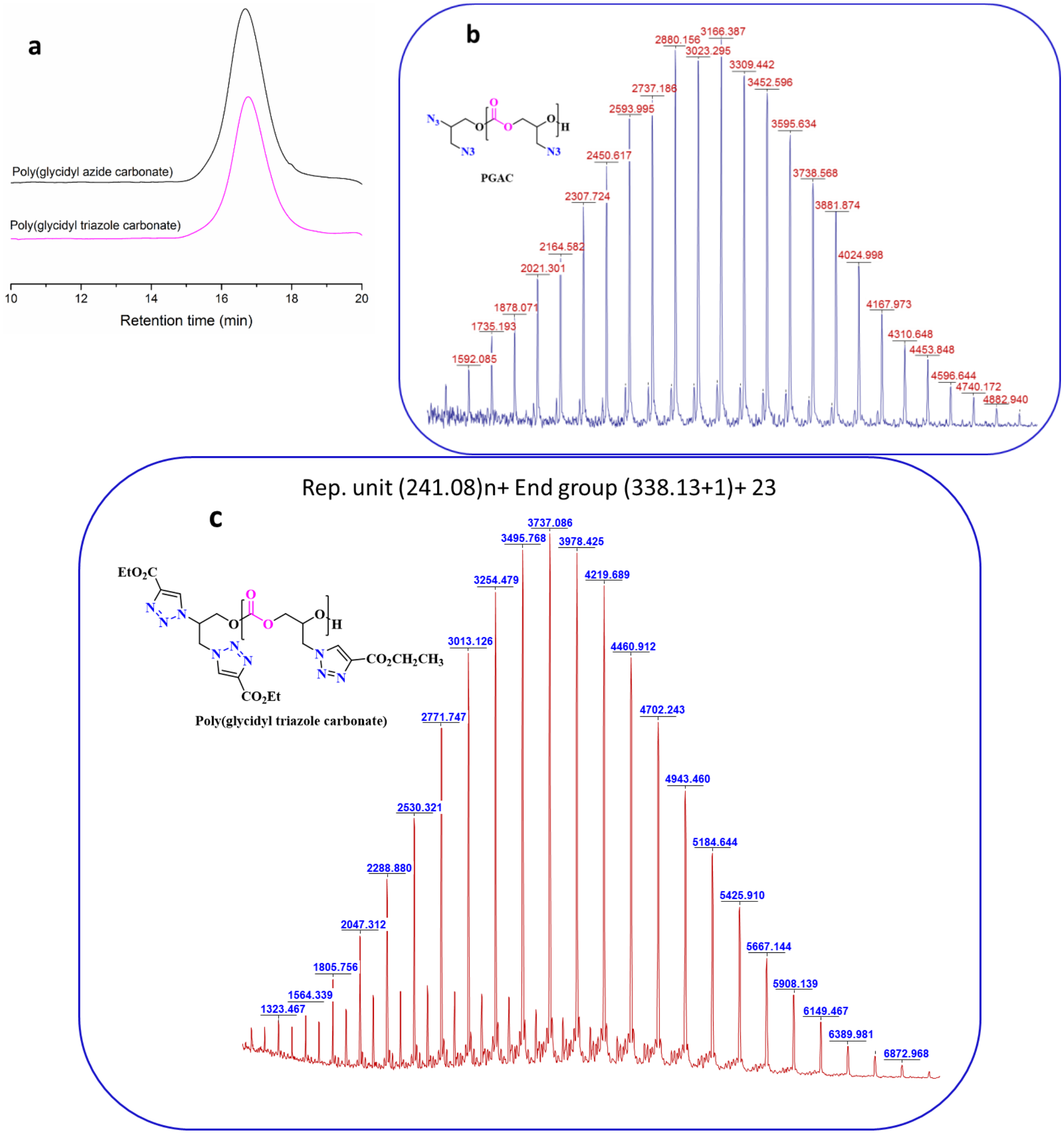


**Supplementary Figure 21. a**: GPC traces of PGAC and poly(glycidyl triazole carbonate) and **b**: MALDI-ToF analysis of PGAC and **c**: poly(glycidyl triazole carbonate). The major mass signals matches perfectly with the end groups as depicted in the above Supplementary Figure c.

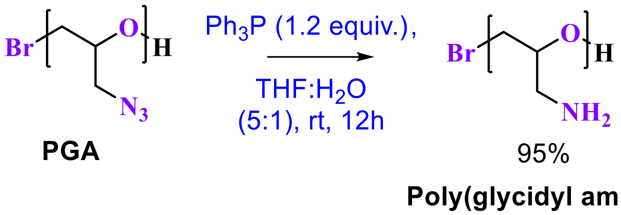


**Supplementary Figure 22**: Reduction of PGA to poly(glycidyl amine) under Staudinger conditions: Reaction conditions: PGA (1 equiv.), $PPh_3$ (1.2 equiv. with respect to azido function), THF:$H_2O$ (5:1) at room temperature, overnight (95%). After the completion of reaction poly(glycidyl amine) forms a separate layer at the bottom. It was collected by precipitation in THF and centrifuged. After removing THF the obtained poly(glycidyl amine) was dried under vacuum and characterized.

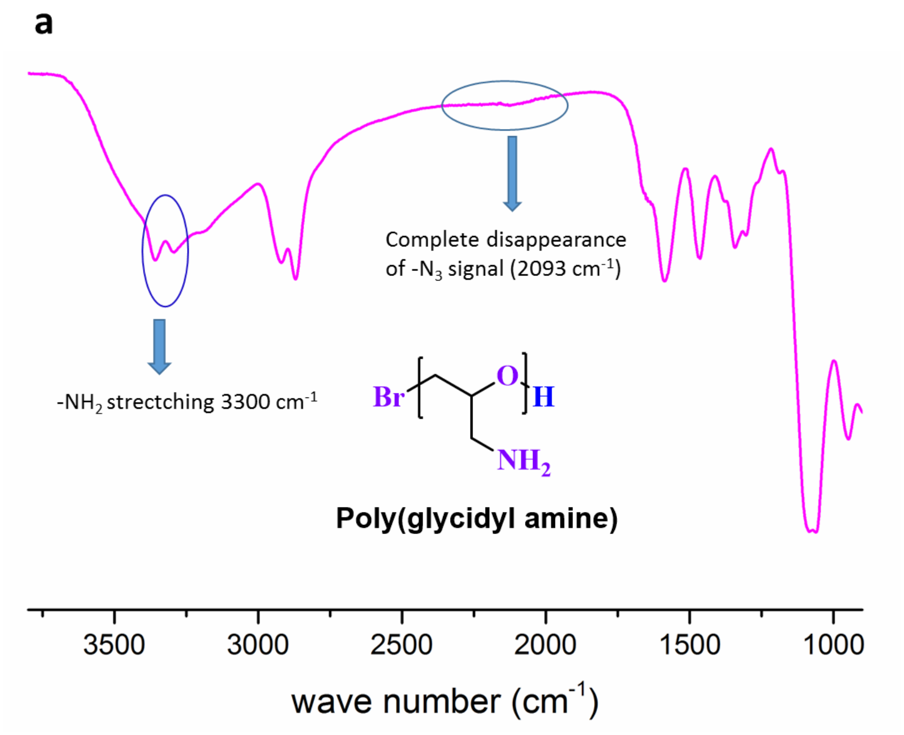

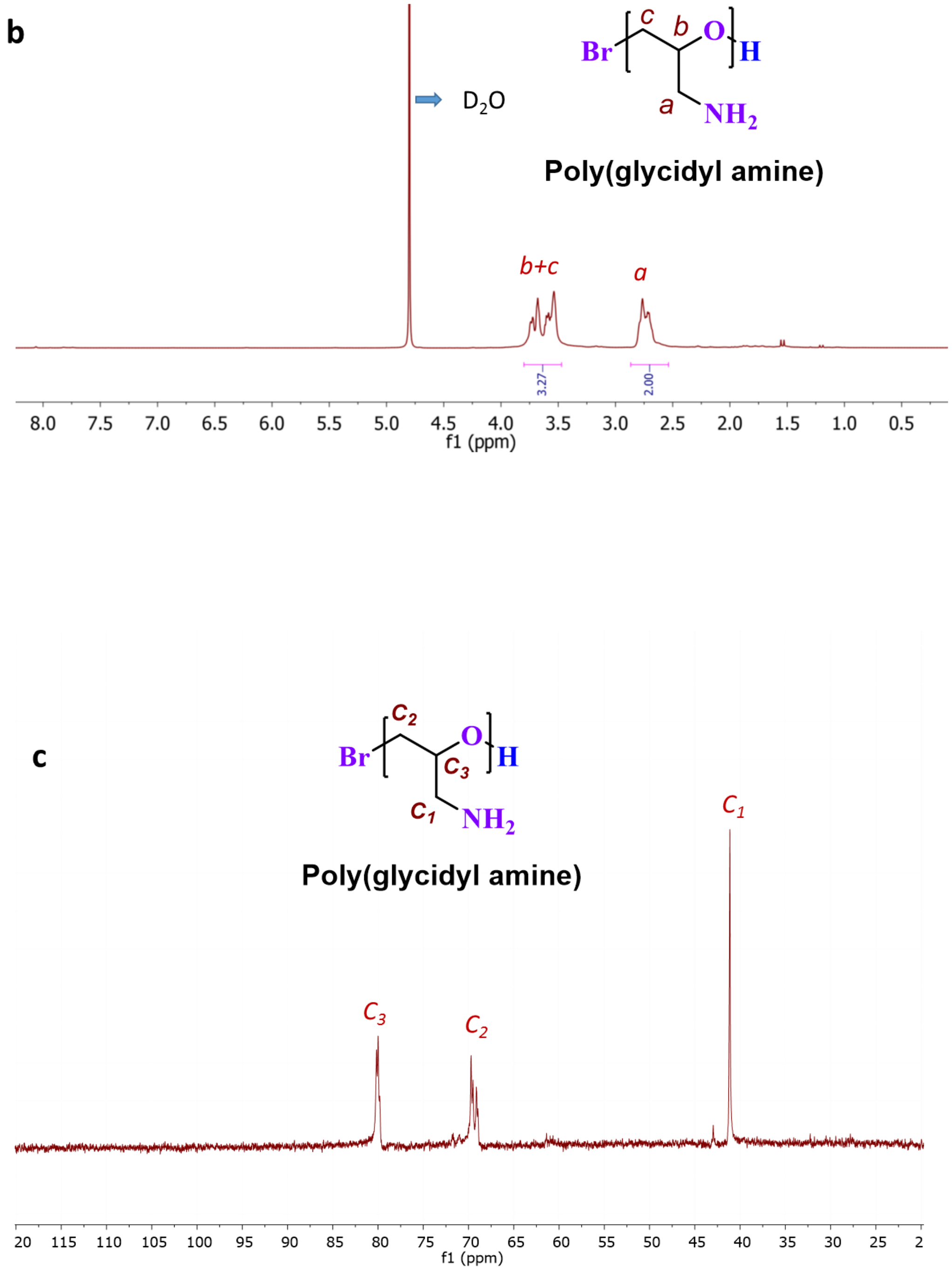


**Supplementary Figure 23**. **a**: FTIR; **b**: $^{1}H$ NMR and **c**: $^{13}C$ NMR spectra of poly(glycidyl amine). The complete disappearance of the azido signal at 2093 cm$^{-1}$ and the appearance of doublet at 3300 cm$^{-1}$ for primary amine (-$NH_2$) in FTIR indicate the reduction of azido to amine functions : an upfield shift of the methylene protons and carbon signal in the NMR spectrum corroborates the complete transformation of azido into amine functions in the polymer backbone.
